# Early motor skill acquisition in healthy older adults: brain correlates of the learning process

**DOI:** 10.1093/cercor/bhad044

**Published:** 2023-03-14

**Authors:** Manon Durand-Ruel, Chang-hyun Park, Maëva Moyne, Pablo Maceira-Elvira, Takuya Morishita, Friedhelm C Hummel

**Affiliations:** Defitech Chair of Clinical Neuroengineering, Neuro-X Institute (INX) and Brain Mind Institute (BMI), École Polytechnique Fédérale de Lausanne (EPFL), Chemin des Mines 9, Geneva 1202, Switzerland; Defitech Chair of Clinical Neuroengineering, Neuro-X Institute (INX) and Brain Mind Institute (BMI), EPFL Valais, Clinique Romande de Réadaptation, Av. Grand-Champsec 90, Sion 1951, Switzerland; Defitech Chair of Clinical Neuroengineering, Neuro-X Institute (INX) and Brain Mind Institute (BMI), École Polytechnique Fédérale de Lausanne (EPFL), Chemin des Mines 9, Geneva 1202, Switzerland; Defitech Chair of Clinical Neuroengineering, Neuro-X Institute (INX) and Brain Mind Institute (BMI), EPFL Valais, Clinique Romande de Réadaptation, Av. Grand-Champsec 90, Sion 1951, Switzerland; Defitech Chair of Clinical Neuroengineering, Neuro-X Institute (INX) and Brain Mind Institute (BMI), École Polytechnique Fédérale de Lausanne (EPFL), Chemin des Mines 9, Geneva 1202, Switzerland; Defitech Chair of Clinical Neuroengineering, Neuro-X Institute (INX) and Brain Mind Institute (BMI), EPFL Valais, Clinique Romande de Réadaptation, Av. Grand-Champsec 90, Sion 1951, Switzerland; Clinical Neuroscience, University of Geneva Medical School, Chemin des Mines 9, Geneva 1202, Switzerland; Defitech Chair of Clinical Neuroengineering, Neuro-X Institute (INX) and Brain Mind Institute (BMI), École Polytechnique Fédérale de Lausanne (EPFL), Chemin des Mines 9, Geneva 1202, Switzerland; Defitech Chair of Clinical Neuroengineering, Neuro-X Institute (INX) and Brain Mind Institute (BMI), EPFL Valais, Clinique Romande de Réadaptation, Av. Grand-Champsec 90, Sion 1951, Switzerland; Defitech Chair of Clinical Neuroengineering, Neuro-X Institute (INX) and Brain Mind Institute (BMI), École Polytechnique Fédérale de Lausanne (EPFL), Chemin des Mines 9, Geneva 1202, Switzerland; Defitech Chair of Clinical Neuroengineering, Neuro-X Institute (INX) and Brain Mind Institute (BMI), EPFL Valais, Clinique Romande de Réadaptation, Av. Grand-Champsec 90, Sion 1951, Switzerland; Defitech Chair of Clinical Neuroengineering, Neuro-X Institute (INX) and Brain Mind Institute (BMI), École Polytechnique Fédérale de Lausanne (EPFL), Chemin des Mines 9, Geneva 1202, Switzerland; Defitech Chair of Clinical Neuroengineering, Neuro-X Institute (INX) and Brain Mind Institute (BMI), EPFL Valais, Clinique Romande de Réadaptation, Av. Grand-Champsec 90, Sion 1951, Switzerland; Clinical Neuroscience, University of Geneva Medical School, Chemin des Mines 9, Geneva 1202, Switzerland

**Keywords:** fMRI, short-term motor learning, healthy aging, parametric modulation, continuous changes

## Abstract

Motor skill learning is a crucial process at all ages. However, healthy aging is often accompanied by a reduction in motor learning capabilities. This study characterized the brain dynamics of healthy older adults during motor skill acquisition and identified brain regions associated with changes in different components of performance. Forty-three subjects participated in a functional magnetic resonance imaging study during which they learned a sequential grip force modulation task. We evaluated the continuous changes in brain activation during practice as well as the continuous performance-related changes in brain activation. Practice of the motor skill was accompanied by increased activation in secondary motor and associative areas. In contrast, visual and frontal areas were less recruited as task execution progressed. Subjects showed significant improvements on the motor skill. While faster execution relied on parietal areas and was inversely associated with frontal activation, accuracy was related to activation in primary and secondary motor areas. Better performance was achieved by the contribution of parietal regions responsible for efficient visuomotor processing and cortical motor regions involved in the correct action selection. The results add to the understanding of online motor learning in healthy older adults, showing complementary roles of specific networks for implementing changes in precision and speed.

## Introduction

Motor learning is a process by which a motor skill is acquired with repeated practice. It is characterized by a succession of stages in which performance increases while functional and structural brain changes occur (Dayan and Cohen 2011). These stages are described as an initial fast learning stage (sometimes referred to as “early online learning”) that occurs within the first minutes of practice of the motor task, followed by a slow learning stage unfolding over multiple days and involving several sessions of practice interleaved with periods of rest (Doyon and Benali 2005). With age, the ability to learn new motor skills is reduced (Brown et al. 2009), and these age-related differences have been associated with different mechanisms such as the degree of task complexity (Voelcker-Rehage 2008; Onushko et al. 2014), a decrease in processing speed (Salthouse 2000; Critchley et al. 2014), or a more general cognitive decline that would impact motor learning (Bo et al. 2009; Bishop et al. 2010; Anguera et al. 2011). Regarding the initial fast learning stage, while several studies reported similar improvements in older compared to younger adults (Seidler 2006; Brown et al. 2009), other reports indicate significant differences (Daselaar et al. 2003; Shea et al. 2006; Zimerman et al. 2013; Maceira-Elvira et al. 2022).

The neural correlates of the initial motor learning acquisition phase have been extensively studied in young adults thanks to functional magnetic resonance imaging (fMRI) (see Dayan and Cohen 2011 for review; Hardwick et al. 2013; Lohse et al. 2014 for meta-analyses). It is now well accepted that the first acquisition of a motor skill relies on 2 different, but interacting networks, namely a cortico-cerebellar and a cortico-striatal network (Hikosaka et al. 2002; Doyon and Benali 2005). Within the cortical correlates, frontoparietal associative areas are thought to be recruited when the spatial coordinates of the motor skill are acquired, a process occurring fast, while sensorimotor areas are involved in the acquisition of motor coordinates, a process occurring on a slower timescale (Hikosaka et al. 2002). In addition to the cortical correlates, subcortical regions, i.e. the cerebellum and the basal ganglia, have been shown to be involved in a cortico-cerebellar and cortico-striatal circuit (Doyon et al. 2003), both recruited in the early motor learning phase. In the aging population, the circuits recruited during motor skill acquisition are similar (Lin et al. 2012; Fogel et al. 2014; Berghuis et al. 2019), but with more widespread patterns of activation and additional bilateral frontal, motor, and temporal areas (Voelcker-Rehage 2008; Turesky et al. 2016; Berghuis et al. 2019). Several cognitive models were proposed in the recent years to explain the compensatory brain mechanisms in the aging population: The Hemispheric Asymmetry Reduction in Old Adults model (Cabeza 2002) states that more bilateral activation in motor and frontal areas allow to reach comparable performance to young adults; the Compensation-Related Utilization of Neural Circuits Hypothesis (Reuter-Lorenz and Cappell 2008) claims that higher neural recruitment of cognitive circuits occurs in older adults; while the Posterior–Anterior Shift (PASA) model (Davis et al. 2008) explains the age-related reduction in activation of posterior brain regions as a manifestation of the impairment in sensory processing that would be compensated by increases in activation of frontal regions.

Most of the studies investigating single-session motor learning usually employ a pre-post design with a practice period performed outside of the MRI scanner (Boe et al. 2012), or compare an already-learned task to a new task (Jenkins et al. 1994). However, averaging activation over blocks may not capture faithfully the dynamics of online motor learning (Gabitov et al. 2015). Especially during the initial motor learning acquisition phase, substantial changes in activity occur in different cortical regions (Toni et al. 1998; Weaver 2015) in young subjects. Within-session dynamic changes were only sparsely studied in young adults (Toni et al. 1998; Floyer-Lea and Matthews 2005; Boe et al. 2012) and older adults (Godde et al. 2018). Furthermore, in most cases, the literature reports time-related brain changes without considering the changes in relation to performance (Godde et al. 2018; Berghuis et al. 2019). The relationship between single-session whole-brain activation and continuous behavioral changes is quite scarce (Orban et al. 2010; Gobel et al. 2011; Choi et al. 2020) and usually includes one component of performance, i.e. either speed or accuracy. Improvement on the finger-tapping task for example, one of the most used tasks in the motor learning field, is generally described in terms of speed (Orban et al. 2010). A few studies however looked at different components of motor performance, but either in young adults (Lefebvre et al. 2012) or on multiple-day learning (Wadden et al. 2013).

Considering the limited amount of reports in regard to within-session brain changes and their relationship to performance during the acquisition of a motor skill in older adults, we designed a task-based whole-brain fMRI study involving a novel motor learning task and assessed time-related and performance-related brain activation patterns during the practice session. The motor learning task is an adapted version of the sequential visual isometric pinch task (SVIPT) (Camus et al. 2009; Reis et al. 2009; Zang et al. 2018), which to our knowledge was not yet investigated with task-based fMRI during the skill acquisition process. We expected to see behavioral improvements on the task as it was shown before that older adults can acquire a motor skill (Rieckmann et al. 2010; Godde et al. 2018; Berghuis et al. 2019). Literature shows that older adults usually favor accuracy over speed (Salthouse 1979; Forstmann et al. 2011), we wondered whether we would observe a similar pattern in the initial learning of this novel motor skill. As for the brain activation patterns evoked by the execution of this task, we expected a similar pattern of activation to the one reported in the motor sequence learning literature involving tracking and force modulation (Sterr et al. 2009; Godde et al. 2018; Berghuis et al. 2019). Especially, we expected that a distributed and wide pattern of cortical regions, cerebellum, and basal ganglia activation would be observed when compared with baseline. As for the patterns of changes throughout time, we expected to observe significant decreases of brain activation as practice advances with less recruitment of frontal areas suggesting less cognitive control (Doyon and Benali 2005), visual areas showing more efficient visual processing (Graydon et al. 2005; Berghuis et al. 2019) paralleled by an increase in activation in parietal and motor-related regions suggesting the formation of motor coordinates (Hikosaka et al. 2002). Furthermore, the cerebellum is thought to be involved in the formation of internal models (Shadmehr and Krakauer 2008); thus, we expected more recruitment in the early phase compared to the late phase of the acquisition. Apart from the dynamics of cortical organization related to the repetition of the task, we investigated the regions specifically involved in the change of performance. Reviewing the literature on performance-related brain activation (Wadden et al. 2013), we expected to observe different neural patterns associated with the different components of performance improvement, i.e. speed and accuracy. As this task was never tested in older adults, the brain–behavior analysis was more explorative. Nevertheless, as the initial training session involves the formation of internal models and spatial coordinates to optimize performance (Sakai et al. 1998; Hikosaka et al. 2002), we expected the cerebellum and frontoparietal areas to play a role in accuracy (less error) and frontoparietal areas involved in sensory processing (Coull et al. 1996; Sakai et al. 1998) for speed.

## Methods

### Subjects

Forty-three healthy right-handed older adults participated in the study (*N* = 27 female, mean age ± SD = 69.5 ± 4.6, age range = 61–80 years old, mean laterality quotient Edinburgh Handedness Inventory = 83.6 ± 20.5; Oldfield 1971). We included subjects with the following inclusion criteria: older than or equal to 60 years old, absence of contraindication for transcranial electric stimulation (tES), transcranial magnetic stimulation (TMS), or magnetic resonance imaging (MRI). These contraindications comprised neuropsychiatric diseases, history of seizures, intake of psychoactive medication that potentially interacts with tES or TMS, pregnancy, and intake of narcotic drugs. Furthermore, we excluded subjects requesting not to be informed in case of incidental findings. The data of *N* = 41 subjects were finally included in the analysis as 2 subjects did not understand well the motor task or had vision difficulties in the MRI scanner. The study was carried out in accordance to the Declaration of Helsinki. Written informed consent was obtained from all subjects. Approval was obtained from the cantonal ethics committee, Geneva, Switzerland (project number: 2017-00224).

### Experimental design

The experiment was designed as a multiple-days study. On Day 0, subjects were screened and were explained the experiment in detail. They filled questionnaires to confirm the absence of MRI, tES, and TMS contraindications as well as to assess the cognitive abilities (the Montréal Cognitive Assessment; Nasreddine et al. 2005), handedness (Edinburgh Handedness Inventory; Oldfield 1971), and quality of sleep (Pittsburgh Sleep Quality Index; Buysse et al. 1989).

**Fig. 1:**
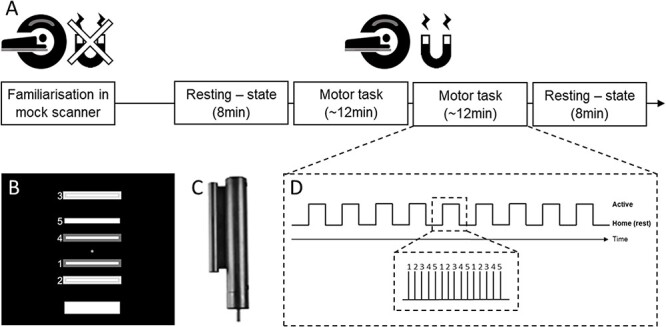
A) Magnetic resonance imaging (MRI) training session. Subjects were familiarized to the task in the mock scanner and were then brought to the MRI environment for resting-state sessions and task-based functional MRI. B) Screen of the sequential grip force modulation task (SGFMT). Subjects navigated a cursor as fast and accurately as possible by modulating their grip force between a home zone and each of 5 numbered target zones following a sequential order. C) MRI-compatible fiber optic grip force sensor used in the present study. D) Block design of one session.

On Day 1, subjects were asked to refrain from drinking caffeinated drinks. After arriving at the lab, the subjects were familiarized to the motor task with standardized explanations and by observing the experimenter performing it (Fig. 1A). They were then asked to practice in a mock scanner for one block in a supine position. The first MRI session comprised one resting-state scan of 8 min followed by 2 sessions of task and ended with one last resting-state scan (Fig. 1A). During the afternoon, subjects underwent a noninvasive brain stimulation sham-controlled intervention associated with a period of sleep. Following the sleep period, follow-up behavioral sessions were performed over multiple days to assess the effect of the stimulation on behavioral improvement. As the main focus of the present study is understanding the neural dynamics during the initial fast learning session, the results of the resting-state scans and of the effects of stimulation will be presented elsewhere.

### Motor learning task

The motor skill learning task consisted of a sequential grip force modulation task (SGFMT) adapted from Reis and colleagues (2009) and from a previous study in the lab (Wessel et al. 2020). It was implemented in Matlab (version R2018a) and displayed in the MRI scanner with a screen behind the head of the subjects who could see it thanks to a tilted mirror above their eyes. The grip forces were sampled with a fiber optic grip force sensor (Current designs, Inc., Philadelphia, PA, USA) compatible with the MRI environment (Fig. 1C). Subjects controlled an onscreen cursor with the grip force sensor using their nondominant left hand. The cursor moved vertically upwards with increasing force while it went back to the initial position at the bottom of the screen when the subject released the gripper. The subjects were asked to navigate the cursor between a home zone and 5 target zones (Fig. 1B) scaled to individual maximal force measured before the start of the task. The topmost bar corresponded to 70% of the maximal force and placed at 85% of the height of the computer screen. The instruction was to place the cursor in each target by following the sequence from 1 to 5 as fast and accurately as possible and by releasing the gripper after reaching each target. When the cursor reached the correct target and was maintained in the target for 200 ms, the success was made aware by the appearance of a white frame on the target. If the cursor stopped for more than 200 ms outside of the correct target, the trial was labeled as being wrong and the failure was notified by the appearance of a dark gray frame (Fig. 1B). Each session of the task consisted of 8 blocks of practice (Fig. 1D) of the learning sequence and 1 block of random sequence placed at the fifth block. Each block was preceded by a countdown from 5 to 1 displayed on the screen. No other starting cues were given and the movements of the cursor were self-paced. Each block terminated when 3 repetitions of a sequence were performed (regardless of accuracy of the movements) and were followed by 15 s of rest indicated by a white cross on a black background.

### Behavioral data analysis

**Fig. 2:**
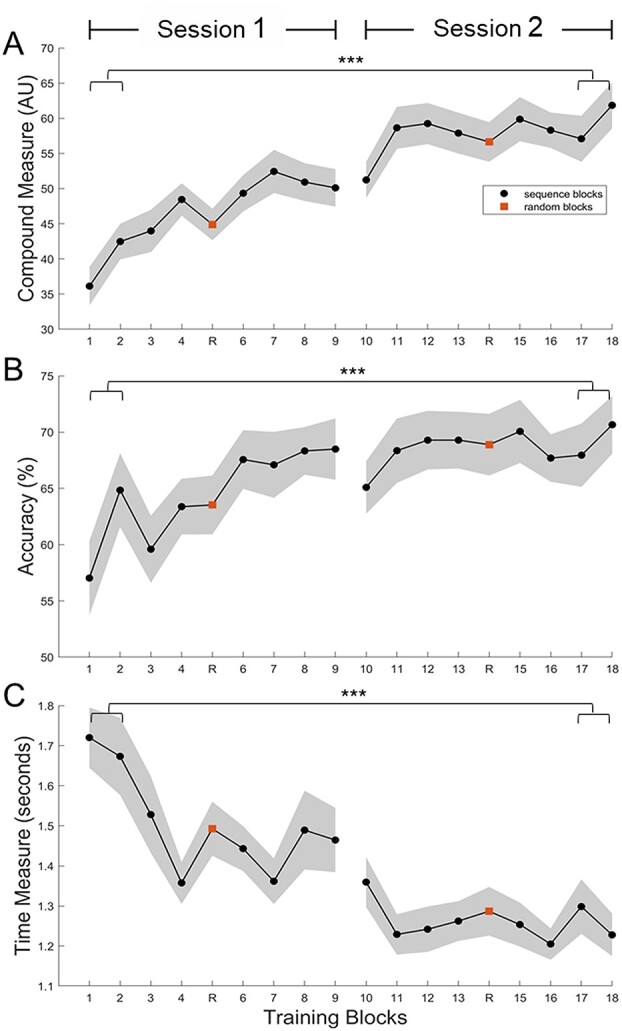
Evolution of performance measures depicted in the form of A) a compound measure of B) accuracy and C) time. The training consisted of 2 learning sessions of 8 blocks of practice of the training sequence and 2 random blocks (depicted in orange in the figure). Shaded areas are the standard error of the mean (SEM). ^*^^*^^*^Statistical significance with *P*-value inferior to 0.001.

The motor performance was first computed in terms of accuracy and average time to reach targets across trials (Fig. 2). When analyzing the behavioral data, we noticed that some trials were invalid because of a limitation of the gripper. These invalid trials (mean percentage of all trials ± SD = 1.8% ± 2.3%, range = 0–8.9%, see the details for each session in Supplementary Table S1) were removed from the analysis. Following this quality check, accuracy was computed for each block as the percentage of correct trials per block. The average time per block was calculated as the mean time to reach each valid trial (the time spent from the moment the cursor left the home zone to the moment the cursor stopped). In order to obtain a single compound score reflecting both speed and accuracy, we used a modified calculation as proposed by Townsend and Ashby (1978), in which we computed the ratio of the accuracy to the average time per block. For the assessment of online learning, we performed a paired samples *t*-test analysis taking the average of the first and last 2 blocks of the training. Normality was tested with Shapiro–Wilk statistical test (Shapiro and Wilk 1965).

### fMRI data acquisition and analysis

Imaging data were acquired with a 3 T Magnetom Prisma scanner (Siemens Healthcare AG, Erlangen, Germany) with a 64-channel coil. Multislice whole-brain T2^*^-weighted functional MRI images were obtained with an interleaved gradient-echo planar imaging of 70 slices (TR = 900 ms TE = 32 ms, FA = 50°, FOV read = 224 mm, receiver bandwidth = 2480 Hz/Px, acceleration factor = 7, and voxel size = 2 mm^3^). A T1-weighted sagittal anatomical brain image was acquired at the end of the first day, using a magnetization-prepared rapid gradient echo (MP-RAGE) sequence consisting of 192 slices (TR = 2,300 ms, TE = 2.96 ms, TI = 900 ms, FA = 9°, FOV read = 256 mm, GRAPPA factor = 2, receiver bandwidth = 240 Hz/Px, and voxel size = 1 mm^3^). For estimating magnetic field inhomogeneities, we additionally acquired a gradient echo field map.

Functional data were preprocessed and analyzed using SPM12 (http://www.fil.ion.ucl.ac.uk/spm/software/spm12/; Wellcome Centre for Human Neuroimaging, University College London, London, UK) implemented in Matlab (version R2018a). The preprocessing comprised the following steps: realignment and correction for magnetic field distortions, coregistration of the mean functional image to the structural T1-image, segmentation of the T1 image into 3 types of brain tissues (cerebrospinal fluid, white matter, and gray matter), warping of these tissues in standard Montreal Neurological Institute space with mutual information affine registration, and normalization of the functional and T1 images by using the deformation parameters computed in the segmentation procedure. The normalization parameters were subsequently applied to the blood oxygenation level-dependent (BOLD) time series, which were finally spatially smoothed using an isotropic 8-mm full-width at half-maximum Gaussian kernel.

Statistical analysis consisted of general linear models that account for fixed and random effects. The subject-level model included all sessions, each of them modeled with block regressors coding for the practiced sequence, for the preparation phase (countdown), and for the random sequence (fifth block). These regressors consisted of box cars convolved with the canonical hemodynamic response function. Global signals of cerebrospinal fluid and white matter and 6 movement parameters were included as covariates of non-interest. Spike regressors derived from thresholding the framewise displacement (FD) signal (Power et al. 2012) at 2 mm were also included. We adopted a liberal threshold for the FD considering the relatively large head movements in older adults (Savalia et al. 2017). With this threshold, an average of 0.3% of scans (mean percentage of all scans ± SD = 0.3% ± 0.51%, range = 0–2.47%) were discarded by including them as regressors of non-interest. High-pass filtering was implemented in the design matrix using a 128-s cutoff period to remove low-frequency drifts from the time series. Serial correlations were estimated using an autoregressive (order 1) model and a restricted maximum likelihood (ReML) algorithm. Separate models were created to assess the time modulation effect (Model 2) and the performance modulation effect (Model 3), including each of them as orthogonalized parametric regressors. Only first-order modulation was considered for the models. The main performance measure used was the compound measure. Separate secondary analyses were performed post hoc to understand whether the brain regions found to be associated with the compound measure contributed differently to accuracy and speed. The other covariates of non-interest were the same as in the first model described above.

To determine significant activation induced by the task during the training session at the subject level (referred to as execution-related activation), a linear contrast tested the main effect of the task relative to baseline by looking at the average activation over the 2 training sessions in Model 1. To assess which brain regions showed changing activation across time (time-modulated activation), we generated separate contrast images testing the main effect of the time-modulated regressor of Model 2 for sessions 1 and 2, as we expected that the dynamics would be different between the 2 sessions of training. Finally, we assessed which brain regions were involved in better performance (performance-modulated activation) by generating one contrast image from the average activation of the performance-modulated regressor across the 2 sessions. These contrasts allowed to generate statistical parametric maps [SPM(T)] at the individual level. The resulting contrast images were entered in a second-level analysis, accounting for intersubject variance and allowing inferences to be made at the population level.

In the second-level analyses of the training session, one-sample *t*-tests were run on the entire sample as subjects. The contrast images computed in the first-level analyses to assess execution-related activation, time-modulated activation, and performance-modulated activation were entered in second-level analyses using one sample *t*-tests. Additional conjunction analyses were carried out to assess the distributional relationship between time-modulated activation and performance-modulated activation. To do so, we computed the one sample *t*-tests of the performance-modulated activation with inclusive or exclusive masks of the time-modulated activation, thresholded at *P* < 0.05 uncorrected, and inversely. For all fMRI results presented in the next section, we adopted a voxel-wise threshold of *P* < 0.001 uncorrected and a cluster-extent based threshold of *P* < 0.05 corrected for multiple comparisons using family-wise error rate. The anatomical automatic labeling (AAL2) atlas (Rolls et al. 2015) was used to label significant regions of activation.

## Results

### Motor learning task

#### Do older adults improve during the motor learning task?

To test whether the initial scores and end-of-training scores of the compound measure were different, we performed a paired sample *t*-test on the average of the first (mean ± SD = 39.3 ± 15.1) and last (mean ± SD = 59.5 ± 18.7) 2 blocks of the compound measure. This analysis showed a significant difference with *t*(40) = −8.05, *P* < 0.001 (Fig. 2). Cohen’s *d* was estimated at −1.26, which is a large effect based on Cohen’s guidelines (Cohen 1992). Secondary analyses also showed significant improvement between initial and end-of-training scores of accuracy *t*(40) = −3.6, *P* < 0.001, Cohen’s *d* = −0.56 and average time to complete trials *t*(40) = 8.1, *P* < 0.001, Cohen’s *d* = −1.27. We tested whether the learning was sequence-specific (Supplementary Information and Supplementary Figs. S1 and S2; Supplementary Table S2). We could not observe a significant behavioral difference between the training and random blocks. However, when looking at the BOLD activation contrasts in Session 2 (end of the training), we observed a significant difference with more activation in cingulate middle areas, supplementary motor area, frontal opercular areas, cerebellar areas, and right primary motor area for the learned sequence while there was more activation in visual areas for the random sequence. These 2 pieces of evidence are divergent; they suggest that there are both sequence-specific and sequence-independent learning occurring during the first acquisition phase.

### fMRI results

#### Which brain regions are involved in the execution of the task?

To assess which brain regions are activated during the initial encoding of the motor learning task, we computed a one-sample *t*-test on the contrast of the average of the 2 learning sessions. This analysis revealed activation in a wide network comprising primary and secondary motor regions, subcortical nuclei, visual, associative, and frontal areas (Supplementary Fig. S3 and Supplementary Table S2).

#### Which brain regions show activation changes during the training session?

**Fig. 3:**
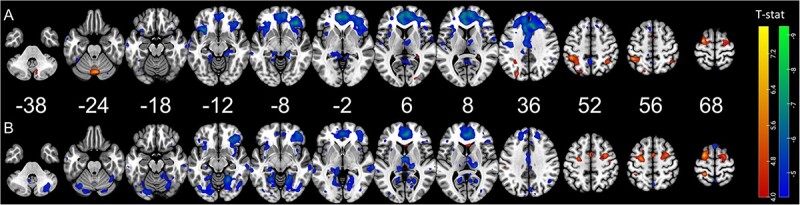
Functional magnetic resonance imaging (fMRI) results of the time-modulated regions during the training sessions with the first session depicted on top (A) and the second session depicted below (B). Blue–green regions have their activation decreasing linearly during the session while red–yellow regions have their activation increasing during the session. Activation maps are depicted at *P*-unc < 0.001.

To investigate the dynamics of brain activation related to the task, we included a regressor modulated by time in the model. One-sample *t*-test was performed individually for each learning session. We observe specific patterns as training advances (Fig. 3). Some regions increase linearly in both sessions, such as the bilateral premotor cortices, contralateral (right) primary motor cortex, and ipsilateral (left) superior parietal lobule (see Table 1A). Other regions decrease linearly in both sessions: contralateral ventromedial prefrontal cortex, bilateral anterior and middle cingulate areas, and bilateral thalami (Table 1B).

**Table 1:** fMRI results of the time-modulated regions during the training sessions with areas showing increasing activation (A) and areas showing decreasing activation (B).

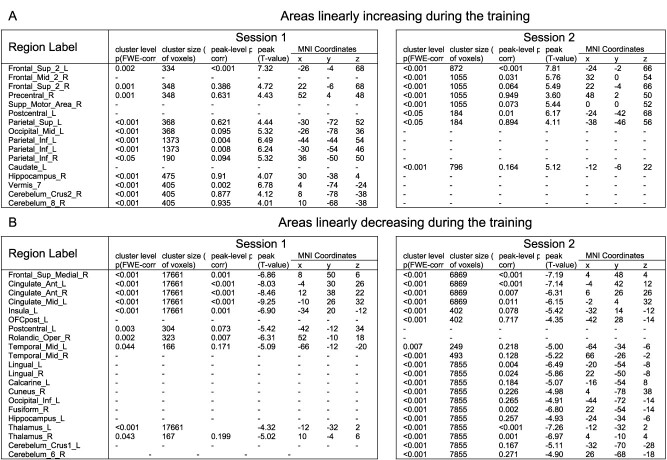

Results are reported at an uncorrected *P* < 0.001 at the voxel level and *P*-FWE < 0.05 at the cluster level. fMRI, functional magnetic resonance imaging; MNI, Montreal Neurological Institute; FWE, family-wise error.

In contrast, activation in some brain areas linearly changes only in the early or the later part of the learning (first vs. second session). In the first training session, we observed increases in bilateral inferior parietal areas, left visual middle occipital area, right hippocampus, right cerebellum, and vermis and decreases in left somatosensory area and right Rolandic operculum. In the second session, the results show increases in the right supplementary motor area, left somatosensory area, and left caudate. We further see decreases in this second session in multiple visual areas and cerebellar areas (Table 1).

#### Brain regions with BOLD activation associated with behavioral change

**Fig. 4:**
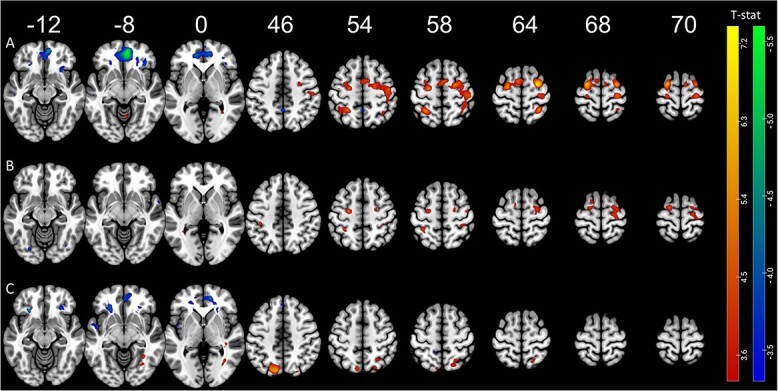
Functional magnetic resonance imaging (fMRI) results of the performance-modulated regions during the training sessions with the regions associated with the compound measure depicted on top (A), the regions associated with accuracy in (B) and the regions associated with average time of trials in (C). In C, the color bars were reversed to be consistent with the other subfigures. Blue–green regions have their activation negatively associated with better performance and red–yellow regions are positively associated. Activation maps are depicted at *P*-unc < 0.001.

To investigate the association between brain activation and behavior, a parametric modulation analysis was performed by including the compound measure per block as a parametric regressor. The results at the group level indicate that areas associated with the improvement of performance (online learning aspect) are bilateral premotor areas, supplementary motor areas, and part of the primary motor and superior parietal areas. In the significant cluster, voxels in the ipsilateral primary motor cortex are significant; the large part of the cluster is, however, located in the contralateral motor areas to the trained hand, as outlined in Fig. 4A. In contrast, areas associated with worse performance comprise frontal and anterior cingulate areas (Fig. 4A and Table 2B).

**Table 2:** fMRI results of the performance-modulated regions during both training with areas positively associated with performance (A) and areas negatively associated with performance (B).

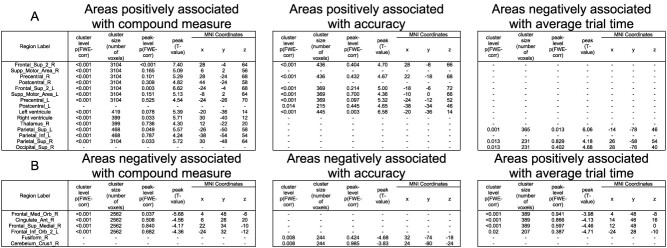

Results are reported at an uncorrected *P* < 0.001 at the voxel level and *P*-FWE < 0.05 at the cluster level. fMRI, functional magnetic resonance imaging; MNI, Montreal Neurological Institute; FWE, family-wise error.

#### Brain regions with BOLD activation associated with speed and accuracy

Speed and accuracy as performance scores have been associated with different neural systems (Wadden et al. 2013; Perri et al. 2014). We aimed to investigate whether this was also the case in the SGFMT. Separate models with each performance measure revealed that the premotor and somatomotor areas were positively associated with accuracy, while activation in frontal cingulate areas and parietal areas was related to time (Fig. 4B and C). More specifically, a longer average time of trials was associated with higher activation in frontal areas and lower activation in bilateral superior parietal areas.

#### Do we observe commonalities and/or differences between time-modulated activation and performance-modulated activation?

As a supplementary analysis, we looked at the conjunction between the practice and compound-related activation. We could observe that most brain regions associated with performance show a linear change in their activation over the course of practice (Supplementary Fig. S4A). The exception was found for the activation of the contralateral postcentral area (S1), which showed a positive association with performance, but did not increase over time (Supplementary Fig. S4B). Inversely, we could observe brain regions, such as visual areas and cerebellar areas, changing over time but were not related to the change of behavior (Supplementary Fig. S5B).

## Discussion

In this study, we examined the neural correlates of short-term online learning of a new motor skill performed in the MRI by healthy older adults. The implementation of the SGFMT was feasible in the MRI environment, and older adults improved significantly on this task during the training sessions, showing that acquisition of the motor skill is possible in our aging cohort. In addition to time-modulated dynamics of brain activation in a wide range of areas of the motor network, we determined here specific brain regions associated with the fast change in performance during the learning process. Worthy of note, we observed regions differentially associated with the change in accuracy or time. Increases in accuracy were associated with increased activation in parts of the cortical sensorimotor network: bilateral primary somatomotor areas and premotor areas. Conversely, decreases in time of execution were related to activation in a frontoparietal network with increased activation in bilateral superior parietal areas and decreased activation in prefrontal and anterior cingulate areas associated with behavioral improvement.

Motor skill acquisition has been extensively studied with 2 types of paradigms: motor adaptation and motor sequence learning (Hardwick et al. 2013; Doyon et al. 2015; Seidler and Meehan 2015; Maceira-Elvira et al. 2022). In the motor sequence learning literature, most studies investigate discrete sequence tasks (Karni et al. 1998; Hikosaka et al. 2002), but this paradigm has recently been critically reviewed (Krakauer et al. 2019) regarding its relevance to daily life activities. In contrast, it was posited that continuous tasks, such as the one used in the present study, are probably more comparable to real-life skills (Reis et al. 2009; Wadden et al. 2013; Choi et al. 2020). In the present study, we show that a cohort of older adults, a population showing impairment in motor performance (Seidler 2006; Voelcker-Rehage 2008; Seidler et al. 2010), can improve significantly on this task. More specifically, although evidence exists regarding the impairment in the precision of force modulation in older adults (Voelcker-Rehage and Alberts 2005), we show that a grip force modulation task could be even learned in a short session by older adults with improvement both in terms of speed and accuracy.

### Activation elicited by the task

This is the first evaluation of the brain activation changes during the learning of the SGMT by means of fMRI; therefore, we first want to discuss the findings in the light of brain activation determined during other motor learning tasks. Consistent with the literature (Sterr et al. 2009; Hardwick et al. 2013; Doyon et al. 2015), a wide network comprising bilateral cerebellum, subcortical areas, and especially basal ganglia and thalamic nuclei, cortical motor, and visual and associative cognitive areas is involved in the acquisition process of the motor skill. It is of note, contrary to what is suggested in the literature implementing the sequential force modulation task (Camus et al. 2009; Reis et al. 2009; Zang et al. 2018), the relevance of the sequence component of this task is not clear. Indeed, we could not observe significant behavioral differences between the random and the learned sequence blocks, suggesting that participants might learn rather sequence-independent aspects of the task. This result is similar to a previous study implementing a highly similar version of the task in young adults (Wessel et al. 2020). This might indicate that unlike other sequence learning tasks such as finger-tapping tasks where the mapping between the action and its consequence is learned quickly (Carment et al. 2018), learning the SGFMT might require a longer learning period for the visuomotor mapping (Sailer 2005). Since the participants were only able to try the task briefly before the initial training, it might be that they learned the visuomotor mapping between the amount of force to apply to control the cursor in the MRI environment. In that sense, the grip force modulation task might be closer to a de novo sensorimotor learning task (Carment et al. 2018; Krakauer et al. 2019). Nonetheless, an additional analysis of the BOLD activation contrasting the sequence and random blocks revealed a differential pattern of activation (especially in the second session of the training) with more activity in visual areas during the random block suggesting that subjects rely more on visual feedback. Inversely, there were more activation in the learned sequence block in middle cingulate areas, cerebellar areas, contralateral primary motor areas, and temporal areas. This pattern of activation is consistent with previous research on motor sequence learning (Jenkins et al. 1994; Daselaar et al. 2003; Bischoff-Grethe et al. 2004). To sum up, the grip force modulation task used in this study seems to involve different learning components, sensorimotor mapping as well as a sequence component.

### Time-related changes in brain activation

Changes in brain activation within a single training session have been studied in young adults (Floyer-Lea and Matthews 2005; Tang et al. 2009; Orban et al. 2010) but, to the best of our knowledge, not in older adults. Our results show similar results to the corpus of literature on young adults. The activation of the cerebellum, a region known to be involved in the early phase of learning when error is high and the movement needs to be corrected quickly (Doyon et al. 2003; Krakauer et al. 2019), first increases followed by decreases in the second session when the accuracy becomes more stable (Fig. 2). This is consistent with the model posited by Doyon et al. (2003), Doyon and Benali (2005), and Doyon et al. (2018), which states that a cortico-cerebellar network is crucial to the early encoding of motor programs. In this model, the researchers present the dynamics of the cortical regions, which consist of constant involvement of motor cortical regions and parietal cortices while they report decreased involvement of hippocampus and frontal associative areas. Our results are partially consistent with this model, as we observe a decrease in the time course of activation of frontal areas and an increase in activation of parietal areas, suggesting that cognitive processes are less needed while procedural processes are increasing as training advances (Sakai et al. 1998). The activation of premotor areas is consistently increasing throughout the training, while the activation of supplementary motor area is especially increasing in the second session. These areas are thought to play a role in the integration of working memory and sensory information for the selection of action (Chen et al. 1995; Hernández et al. 2002; Floyer-Lea and Matthews 2005; Tang et al. 2009). Differently to the model of Doyon, we find substantial decreases in the visual system, especially in the second session. This observation suggests more efficient visuospatial processing at the end of the training as in a report of Berghuis et al. (2019) and stresses the difference between discrete learning tasks and continuous tasks with visual feedback where sensorimotor integration is a crucial component of the learning process, comparable to tracking tasks (Sterr et al. 2009; Carment et al. 2018). Finally, one unexpected result was a consistent decrease of activation in the thalamus observable in both sessions of the training, which is rarely described. This result is probably associated to the presence of motor fatigue as suggested recently (Hou et al. 2016). One interesting aspect to point out in this analysis is that in contrast to other studies that assessed pre-post changes (Floyer-Lea and Matthews 2005; Boe et al. 2012), we assessed the within session changes occurring in the brain while subjects performed the task. In summary, we demonstrated dynamic changes toward decreases in cognitive and visual areas and increases in associative and motor areas during the initial acquisition of a motor learning task.

### Performance-related brain activation

In addition to looking at the overall changes in a single training, we investigated the relationship between activation and performance changes throughout the training. We observed that contralateral primary motor, bilateral secondary motor and somatosensory areas, and bilateral superior parietal areas were positively associated with better performance, while medial frontal and anterior cingulate areas were negatively associated. As for the positive association, previous research reports similar results in finger-tapping tasks (Orban et al. 2010, 2011; Albouy et al. 2012; Gabitov et al. 2015) and in tracking tasks (Kranczioch et al. 2008; Sterr et al. 2009). The association between performance and cerebellar activation suggested in several studies (Orban et al. 2010; Albouy et al. 2012; Wadden et al. 2013) is not clear in the present study. This differential result might be explained by the fact that in the second session of training, despite the fact that performance continues to increase, a decrease in cerebellar activation was observed. It might be that the cerebellum was strongly implicated in error correction leading to improvement of performance in the first session, but not in the second, when errors were already reduced and the improvement in performance was relying on other mechanisms, such as speed improvement. The cerebellum is thought to be involved in the generation of internal models (Shadmehr and Krakauer 2008), which would be corrected at the early stages of training in order to reduce error. It could be that during the second session, the internal model is rather accurate, thus leading to decreased involvement of the cerebellum. We also found negative modulation with performance in medial prefrontal and cingulate areas. These areas are known to be engaged in cognitive processes and effort (Devinsky et al. 1995; Pessiglione et al. 2018) indicating that poorer performance led to increased effort. Most of the above-mentioned areas were present in time-related and performance-related activation (Supplementary Fig. S4). The somatosensory area, however, was modulated by better performance but not by time. A recent study has shown that the contralateral somatosensory cortex is involved in motor planning in order to achieve better movement control (Ariani et al. 2022). In our context, we hypothesize that, although activation in the somatosensory cortex did not change due to the sensory stimulus staying constant, higher activation in the area resulted in better motor planning and thus better performance. Inversely, we could observe that activation in insula, visual, temporal, and lateral frontal areas was decreased over time but was not related to motor performance. This suggests that these areas are decreasing due to the effect of repetition, but their change is not strongly associated with the motor behavioral improvement.

Usually, the improvement on motor sequence learning tasks is assessed in terms of changes in speed rather than accuracy, as accuracy ceils relatively quickly (Boutin et al. 2013; Fitzroy et al. 2021). However, this did not occur in the present task (see Fig. 2), and it allowed to investigate whether different brain areas were involved in these specific aspects of motor performance. Similarly to Wadden and colleagues (2013), who employ a joystick-tracking task, we could disentangle different networks of brain activation related to the time to complete the task and the accuracy while performing the task. Improvement in accuracy was related to premotor and supplementary motor areas, whereas improvement in time was associated with higher activation in parietal areas and inversely related to medial frontal and anterior cingulate areas. Good accuracy in the present task involves selecting a good timepoint to stop increasing grip force; this is consistent with the view that the premotor cortex and supplementary motor area are involved in the temporal control of movement (Halsband et al. 1993). Additionally, the involvement of somatosensory areas in the accurate maintenance of force has been reported before (Mayhew et al. 2017). Lower time to complete trials (better performance) has been suggested to be associated with effective visuomotor processing implemented in parietal areas (Coull et al. 1996; Grefkes et al. 2004). Indeed, the superior parietal lobule is thought to act as a sensory-motor hub for the interaction with external environment (Passarelli et al. 2021) and has been shown to play a role in the rapid processing of visual information in particular (Coull et al. 1996). Inversely, when the time to reach a target increases, it implies that a sustained effort is made by the subject, and thus, the anterior cingulate areas get more involved. This area has been proposed as a region responsible for the online detection of processing conflicts that will lead to deteriorating performance (Carter et al. 1999). In other words, its activation reflects the level of conflict present in the response system. If the time to complete the task is high, it means that the initial representation of directing the cursor is wrong, and thus, the attention needs to be allocated to correct this wrong representation; the anterior cingulate areas might be the region responsible for evaluating this conflict. These different patterns of activation between frontal and posterior areas are consistent with the interpretation of the PASA model (Davis et al. 2008), with older adults showing higher frontal activation compensating for reduced activation in parietal areas involved in sensory processing (Grefkes et al. 2004). In the present experiment, we observed that participants having efficient sensory processing revealed by better performance had more activation in posterior areas and lower activation in frontal areas. The interpretation of whether it is a sole correlate of aging has to be taken with caution, as in the present study there is no young control group. This is a limitation of the study, although the present results are discussed in the context of the existing literature in healthy young adults. Nonetheless, we believe that our brain–behavior results are meaningful on their own to understand how older adults reach good performance during the initial acquisition process. Better performance was achieved by the interplay of distributed brain regions responsible for efficient visuomotor processing and correct selection of action in our cohort of older adults. It is worth noting that common regions, such as the cerebellum and the basal ganglia, usually involved in a good performance on motor sequence learning tasks (Halsband and Lange 2006; Lefebvre et al. 2012; Wadden et al. 2013), were not clearly associated with performance in this study. This discrepancy might be due to the difference in the motor learning task used; indeed, it was posited that the basal ganglia are involved in the organization of individual elements into a sequence and to the automaticity of the execution of this set of actions (Krakauer et al. 2019). As the present task is continuous and seems to involve a dominant visuomotor aspect, the relevance of the basal ganglia might not be so prominent. An additional explanation might be that this initial training period failed to induce a shift from an allocentric spatial strategy to an egocentric-motor one (Hikosaka et al. 2002; Albouy et al. 2013), thus not (yet) involving the basal ganglia in a relevant way in the production of good performance. This latter interpretation is consistent with recent studies involving a force modulation task similar to our own (Godde et al. 2018; Berghuis et al. 2019).

## Conclusion

This work evaluated online learning and brain–behavior correlates during the acquisition of a novel motor learning task in older adults. Spatial precision was associated with higher activation in motor-related cortical areas responsible for action selection, whereas the speed of execution was related to associative areas involved in visuomotor processing. These results show the relevance of continuously monitoring brain activation changes during the acquisition phase of motor learning to understand which brain areas are recruited and associated with better behavior. Furthermore, this work adds to the understanding of underlying processes during motor learning in older adults and paves the way for characterizing potential targets for interventional approaches for older subjects or patients with motor deficits.

## Funding

This research was partially funded by the Defitech Foundation (Morges, CH), the Wyss Center for Bio and Neuroengineering (WP024, WP030; Geneva, CH), the Schmidt-Heiny Foundation (Geneva, CH), by ERA NET NEURON (Discover project, local funding agency Swiss National Science Foundation (SNSF)), and the “Personalized Health and Related Technologies (PHRT-#2017-205)” mechanism of the ETH Domain.

*Conflict of interest statement*: The authors report no competing interests.

## Data availability statement

The data related to this article are available upon reasonable request from the corresponding author.

## CRediT author statement

Manon Durand-Ruel (Conceptualization, Data curation, Formal analysis, Investigation, Methodology, Visualization, Writing—original draft), Chang-hyun Park (Formal analysis, Investigation, Methodology, Supervision, Visualization, Writing—review and editing), Maëva Moyne (Investigation, Methodology, Writing—review and editing), Pablo Maceira-Elvira (Formal analysis, Methodology, Writing—review and editing), Takuya Morishita (Conceptualization, Data curation, Formal analysis, Investigation, Project administration, Supervision, Writing—review and editing), Friedhelm Hummel (Conceptualization, Funding acquisition, Methodology, Project administration, Resources, Supervision, Writing—review and editing).

## Supplementary Material

Supplementary_Material_final_bhad044
