# Supplementary material for: Early motor skill acquisition in healthy older adults: brain correlates of the learning process
Source: Cereb Cortex. 2023 Mar 14;33(12):7356–68. doi: 10.1093/cercor/bhad044 (PMC10267624; doi:10.1093/cercor/bhad044)
Supplement: Supplementary_Material_final_bhad044 [file supplementary_material_final_bhad044.docx]

Supplementary material: Early motor skill acquisition in healthy older adults: brain correlates of the learning process

**Supplementary Information**

Investigation of the difference of trained sequence versus random sequence

Behavior: The random blocks (B5 and B14) did not significantly differ from the neighboring blocks, B4 vs. B5 t(40) = 1.75, p = 0.088, Cohen’s *d* = 0.27, B6 vs. B5 t(40) = 1.75, p = 0.088, d = 0.27, B13 vs. B14 t(40) = 0.49, p = 0.627, Cohen’s *d* = 0.076, B15 vs. B14 t(40) = 1.42, p = 0.163, d = 0.22, although a trend is present for the first random block. This implies that sequence-independent learning was present.

BOLD activation: Although the behavioral results did not show any significant difference between random blocks and neighboring blocks, we checked as a control analysis whether BOLD activity would be different between these blocks to make clear whether there was brain activation specific to the learned sequence. To do so, we implemented a new GLM design at the subject level, creating new regressors for the neighboring blocks. We then computed the contrasts preblock- randomblock, postblock-randomblock for session 1 and 2, with the preblock being B4 or B13 and postblock being B6 or B15. Additionally, we computed the contrast postblock-preblock as a control analysis to see if there was a difference between the neighbouring blocks of the random. We can see the results in the Supplementary Table 1 and the Supplementary Figure 1. As the random block occurs only once within each session, the statistical power is low and we should thus interpret these results with caution. We would expect to see the most differences in the contrast preblock-randomblock in session 2 as the sequence has been learned already for 11 previous blocks. The postblock-randomblock contrast is less of interest as the activity of the random block might have aftereffects. As expected we observed significant differences in the contrast preblock-randomblock in session 2 with more activity during the learned sequence in middle cingulate area, supplementary motor area, frontal opercular areas, cerebellar areas and right primary motor area. Inversely, we observe more activity during the random block in left visual and superior parietal areas. This analysis points toward the fact that although we do not observe a significant difference behaviorally, there is a sequence-specific learning component occurring in the first acquisition phase.

**Supplementary Figures and Tables**

| *Over 41 participants* | All Training | Session 1 | Session 2 |
| --- | --- | --- | --- |
| Mean percentage of all trials | 1.84% | 2.78% | 0.90% |
| Standard deviation | 2.30% | 4.06% | 1.47% |
| Range | 0 - 8.89% | 0 – 16.3% | 0 – 7.41% |

Supplementary Table 1. Summary table for the amount of invalid trials removed during the training session.

Supplementary Figure 1. Differences between the compound behavioral measure of the random blocks (B5 and B14) compared to the neighboring blocks (B4, B6, B13, B15). The error bars are standard deviation from the mean. No differences were significant.


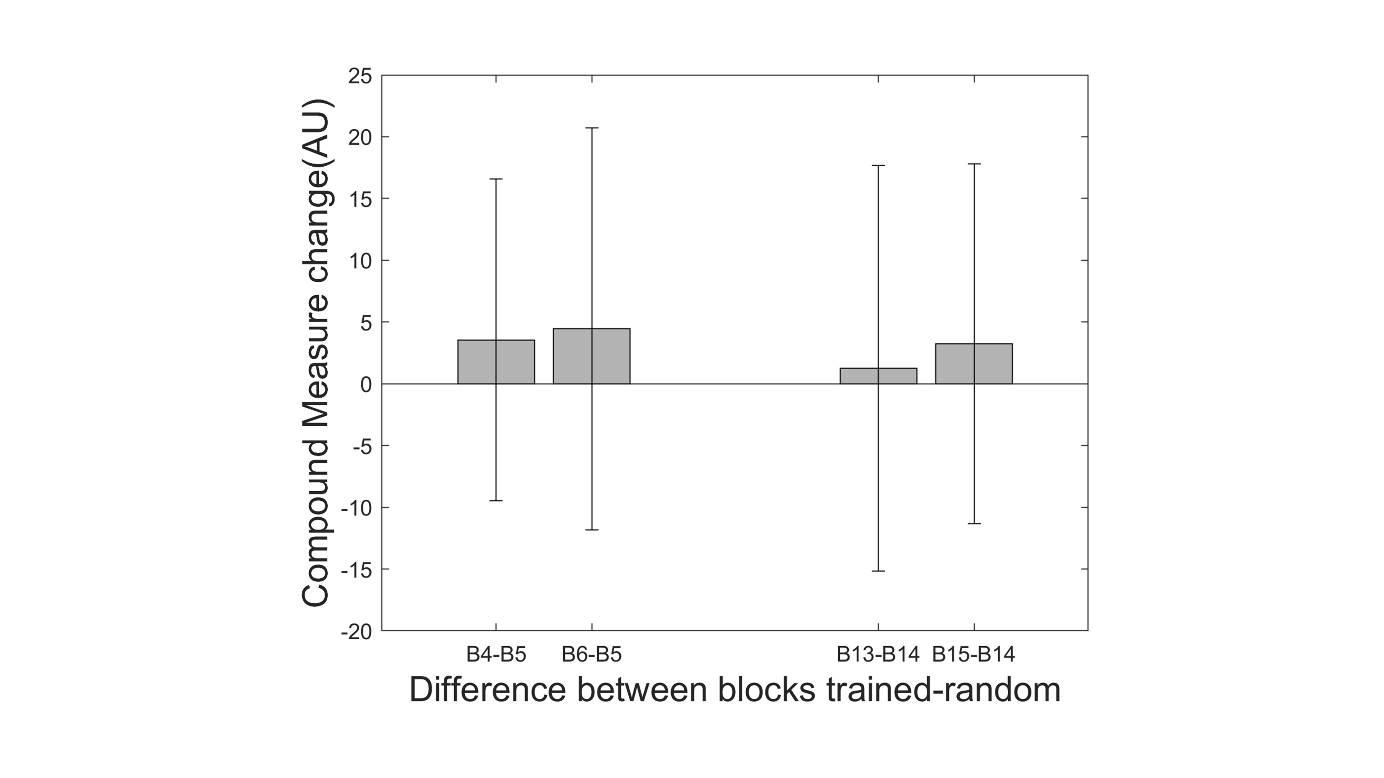

Supplementary Table 2. BOLD activation results for the contrasts between the learned sequence blocks versus the random sequence blocks. (A) Contrast preblock-randblock of Session 1 and Session2. (B) Contrast postblock-randblock of Session 1 and Session 2. Results are reported at uncorrected p<0.001 at the voxel level, cluster level p-FWE<0.05.

***
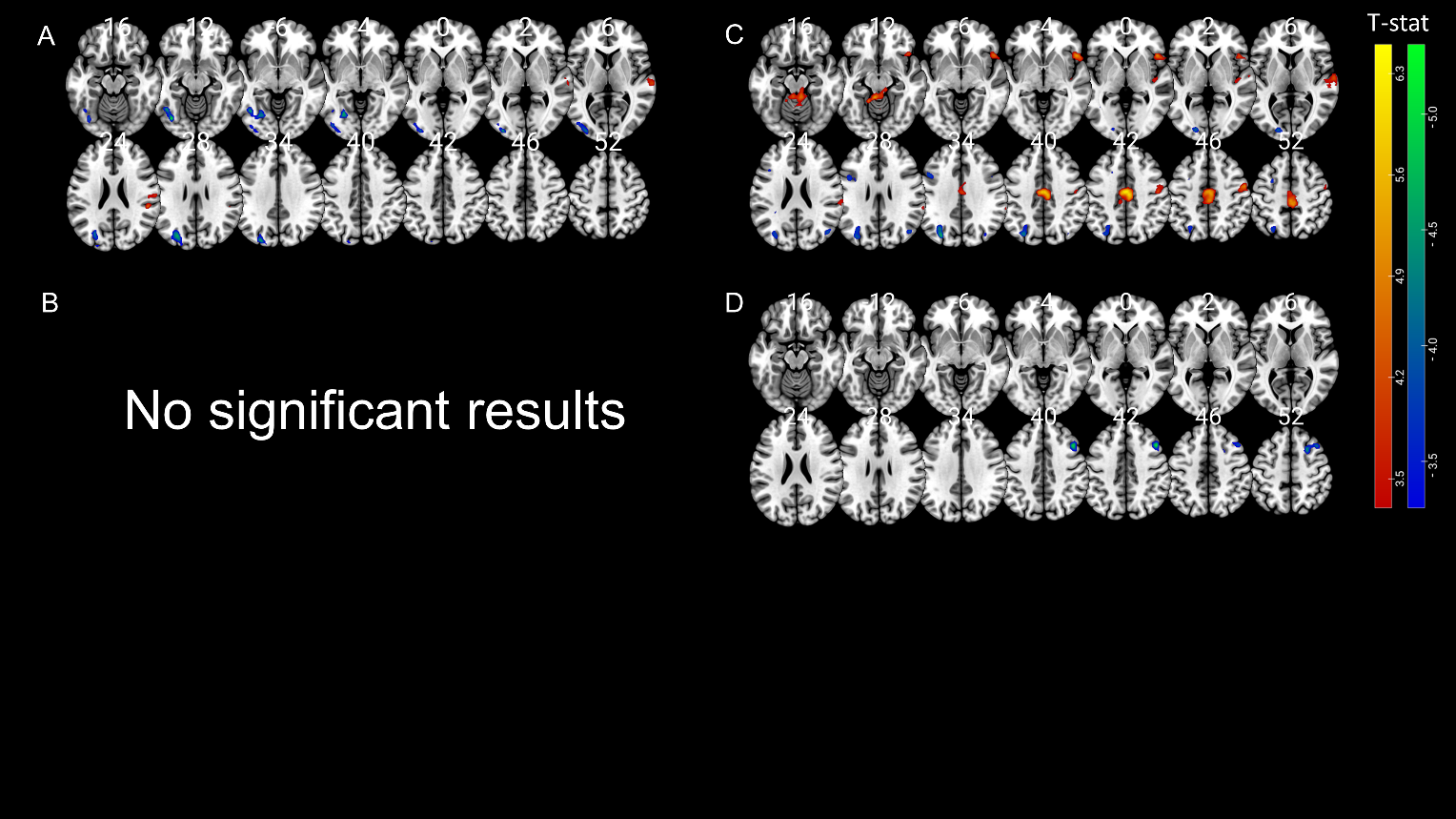
***

Supplementary Figure 2. BOLD activation results for the contrasts between the learned sequence blocks versus the random sequence blocks. The organization of the figure is similar to supplementary Table 1. (A) Contrast preblock-randblock of Session 1. (B) Contrast postblock-randblock of Session 1. (C) Contrast preblock-randblock of Session 2. (D) Contrast postblock-randblock of Session 2. Results are reported at uncorrected p<0.001 at the voxel level, cluster level p-FWE<0.05.

| Region Label | cluster level p(FWE-corr) | cluster size (number of voxels) | peak-level p(FWE-corr) | peak (T-value) | MNI Coordinates | | |
| --- | --- | --- | --- | --- | --- | --- | --- |
|  |  |  |  |  | x | y | z |
| **Main effect of training** | | | | | | | |
| Frontal_Mid_2_R | <0.001 | 47720 | <0.001 | 19.73 | 36 | -8 | 52 |
| Vermis_8 |  |  | <0.001 | 18.28 | -2 | -62 | -30 |
| Precentral_R |  |  | <0.001 | 16.67 | 32 | -20 | 48 |
| Cerebelum_8_R |  |  | <0.001 | 16.51 | 10 | -70 | -44 |
| Frontal_Sup_2_L |  |  | <0.001 | 16.45 | -24 | -6 | 56 |
| Supp_Motor_Area_L |  |  | <0.001 | 16.33 | -10 | -12 | 70 |
| Precentral_R |  |  | <0.001 | 16.27 | 36 | -16 | 52 |
| Occipital_Mid_R |  |  | <0.001 | 16.11 | 32 | -86 | 10 |
| Frontal_Sup_2_R |  |  | <0.001 | 15.93 | 22 | -4 | 60 |
| Parietal_Sup_L |  |  | <0.001 | 15.77 | -20 | -66 | 58 |
| Cerebelum_6_L |  |  | <0.001 | 15.69 | -26 | -56 | -22 |
| SupraMarginal_R |  |  | <0.001 | 15.51 | 46 | -34 | 44 |
| Precentral_R |  |  | <0.001 | 15.50 | 28 | -2 | 50 |
| Occipital_Mid_L |  |  | <0.001 | 15.36 | -28 | -88 | 12 |
| Occipital_Mid_R |  |  | <0.001 | 15.28 | 28 | -72 | 30 |
| Putamen_L | <0.001 | 548 | <0.001 | 11.86 | -24 | -14 | 8 |
| Thalamus_L |  |  | <0.001 | 9.27 | -16 | -16 | 10 |
| Frontal_Mid_2_L | 0.02 | 263 | 0.002 | 6.56 | -40 | 34 | 30 |

Supplementary Figure 3. Rendered figure of the average BOLD activation during the training blocks.


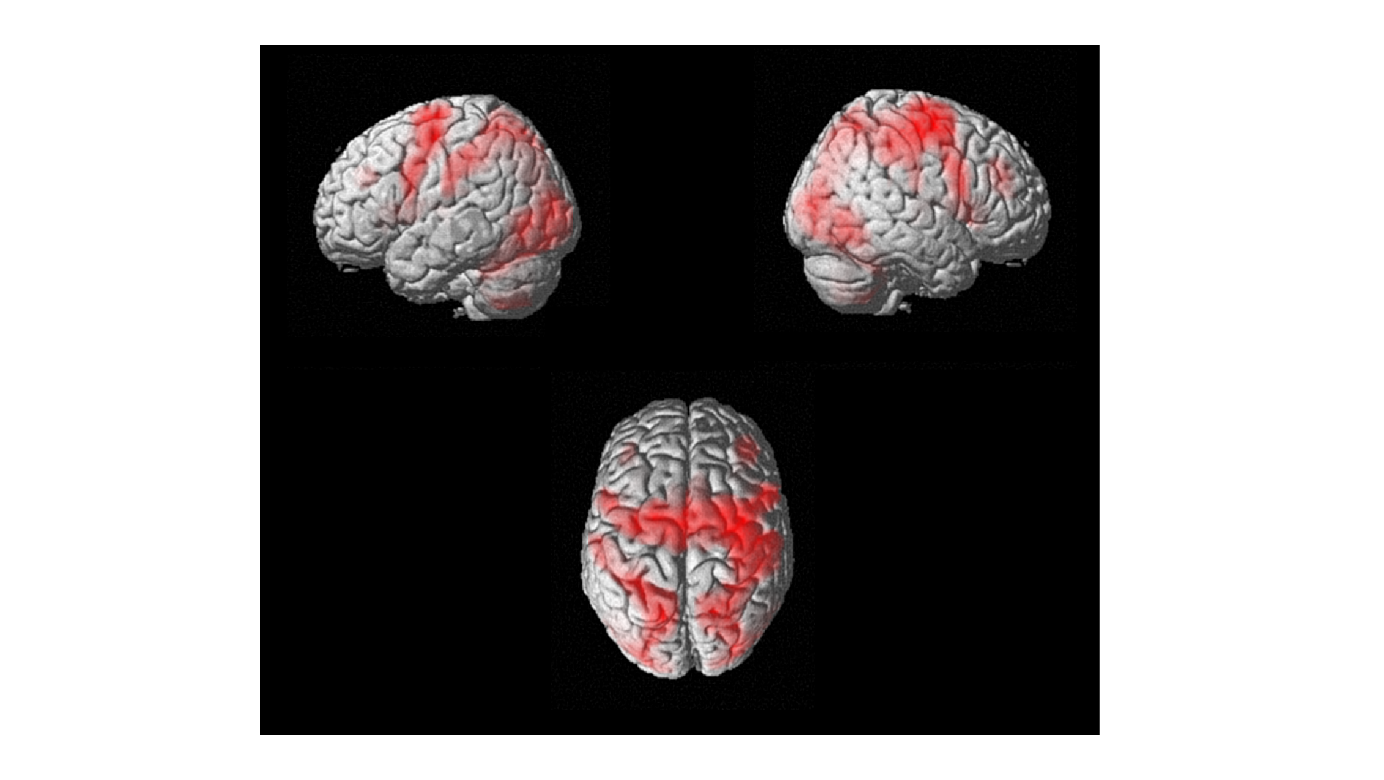


Supplementary Table 3. BOLD activation result for the contrast Average of both training sessions. The main areas are listed. The threshold was at the cluster-level p<0.05 FWE-corrected.

Supplementary Figure 5. Time-modulated brain activation masked by the performance-modulated activation. (A) Time-modulated activation that also showed performance-modulation. The results were computed within an inclusive mask of performance-modulated activation. (B) Regions showing time-related activation which do not show an association with behavior.


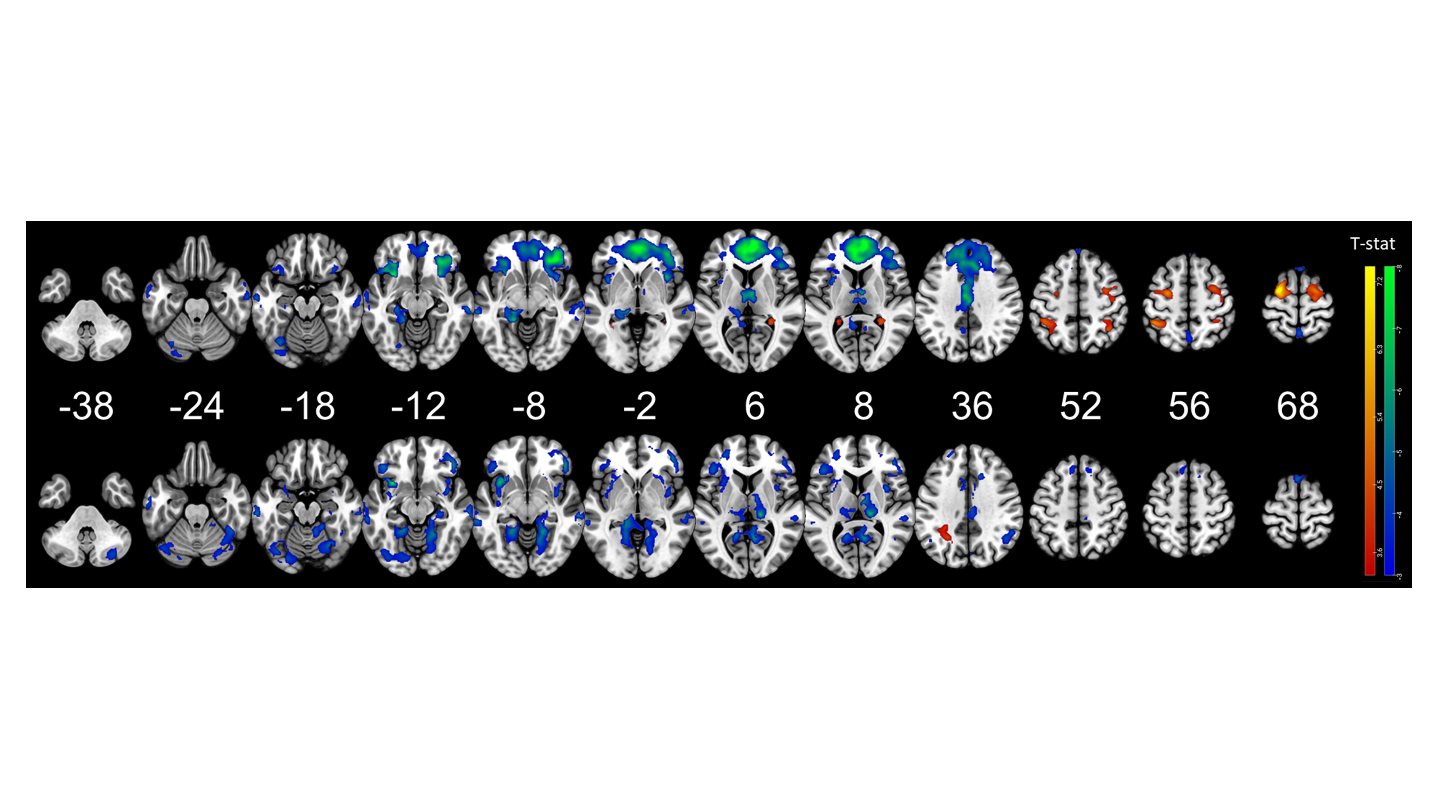


A

B

Supplementary Figure 4. Performance-modulated brain activation masked by the time-modulated activation. (A) Performance-modulated activation that also showed time-modulation. The results were computed within an inclusive mask of time-modulated activation. (B) Regions showing performance-related activation which do not show a linear increase (exclusive mask of time-modulated activation).


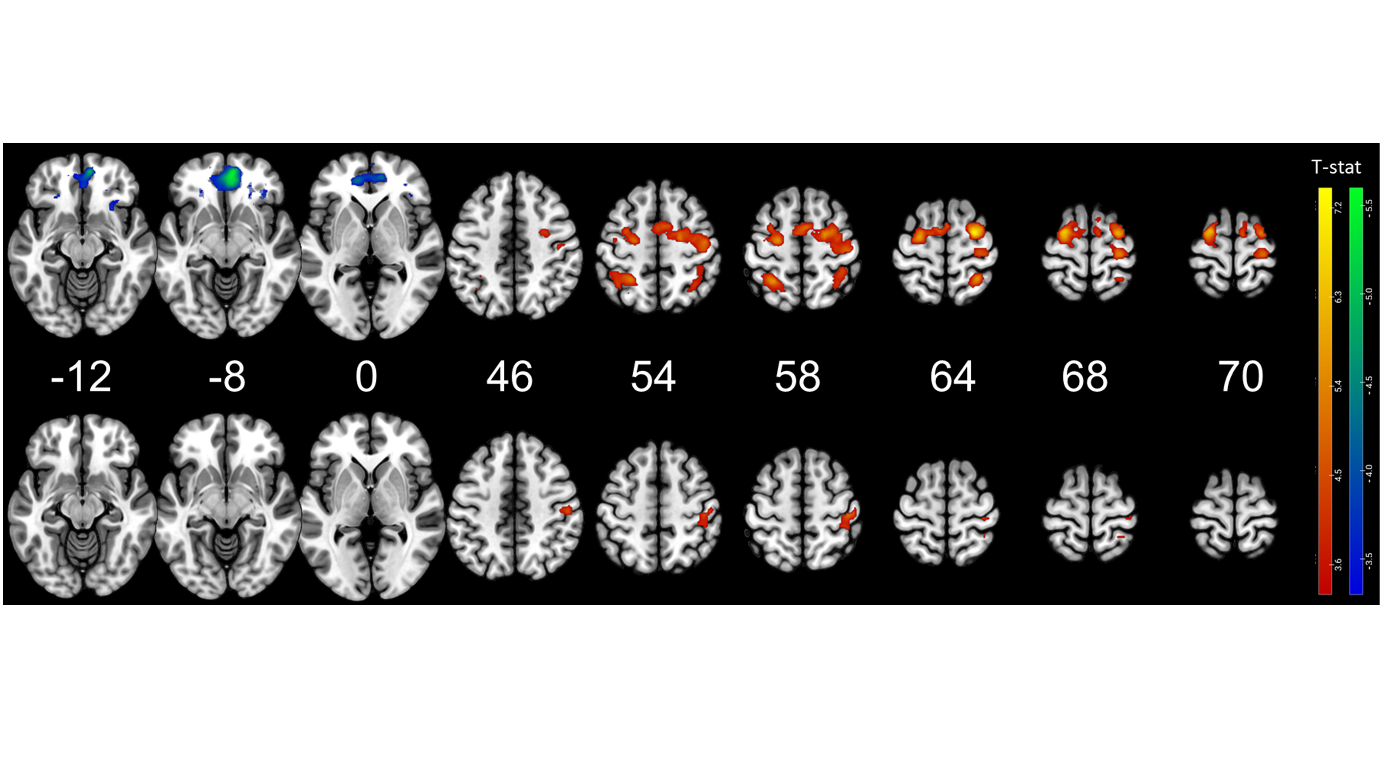


A

B
